# Updates in Immunotherapy for Pancreatic Cancer

**DOI:** 10.3390/jcm13216419

**Published:** 2024-10-26

**Authors:** Robert Connor Chick, Timothy M. Pawlik

**Affiliations:** Department of Surgery, The Ohio State University Wexner Medical Center, Columbus, OH 43210, USA; robert.chick@osumc.edu

**Keywords:** pancreatic cancer, immunotherapy, personalized, tumor microenvironment, immune checkpoint inhibitor, cancer vaccine

## Abstract

Pancreatic ductal adenocarcinoma (PDAC) is an aggressive malignancy with limited effective therapeutic options. Due to a variety of cancer cell-intrinsic factors, including KRAS mutations, chemokine production, and other mechanisms that elicit a dysregulated host immune response, PDAC is often characterized by poor immune infiltration and an immune-privileged fibrotic stroma. As understanding of the tumor microenvironment (TME) evolves, novel therapies are being developed to target immunosuppressive mechanisms. Immune checkpoint inhibitors have limited efficacy when used alone or with radiation. Combinations of immune therapies, along with chemotherapy or chemoradiation, have demonstrated promise in preclinical and early clinical trials. Despite dismal response rates for immunotherapy for metastatic PDAC, response rates with neoadjuvant immunotherapy are somewhat encouraging, suggesting that incorporation of immunotherapy in the treatment of PDAC should be earlier in the disease course. Precision therapy for PDAC may be informed by advances in transcriptomic sequencing that can identify immunophenotypes, allowing for more appropriate treatment selection for each individual patient. Personalized and antigen-specific therapies are an increasing topic of interest, including adjuvant immunotherapy using personalized mRNA vaccines to prevent recurrence. Further development of personalized immune therapies will need to balance precision with generalizability and cost.

## 1. Introduction

Pancreatic ductal adenocarcinoma (PDAC) is an aggressive malignancy with a high annual case fatality rate [1]. Although far less common than breast, prostate, colon, and lung cancer, PDAC is the third leading cause of cancer death in the United States [1]. Routine screening is not recommended in the general population, and many patients present with advanced disease. Unfortunately, even among patients with localized disease at presentation, recurrence after curative-intent resection is common. Fluorouracil- or gemcitabine-based regimens remain the cornerstone of systemic chemotherapy [2,3]. In addition, several randomized trials have demonstrated the clinical benefit of neoadjuvant chemotherapy with or without radiation [4,5,6,7,8,9]. Neoadjuvant chemotherapy has several goals, including early delivery of systemic therapy, testing of disease biology, local downstaging, and treatment of occult metastases.

PDAC generally does not respond well to traditional cytotoxic systemic chemotherapy. Over the last decade, immune therapy has become a pillar of systemic therapy for some other cancer types. Immune checkpoint inhibitors are now widely used with the goal of “releasing the brakes” on the adaptive immune system, allowing tumor-infiltrating lymphocytes (TILs) within the tumor microenvironment (TME) to exert antitumor immune responses. Randomized trials have demonstrated a benefit of immune checkpoint inhibitors in more immunogenic cancers, such as melanoma, clear cell renal cell carcinoma, and some lung cancers, as well as other less immunogenic cancers such as esophageal, gastric, hepatocellular, and biliary tract cancers. Though melanoma, for example, has a high tumor mutational burden (TMB) and a more inflamed and immune-enriched TME, PDAC has an immune privileged TME marked by dense desmoplastic stroma and a paucity of immune cells [10]. Interestingly, standard chemotherapy and/or radiation can alter the TME to make it more immunogenic, but this effect is not sufficient for long-term antitumor immunity [11,12,13,14].

To date, few clinical trials have demonstrated a meaningful benefit of currently available immune therapies in the setting of PDAC. More recently, several investigators have attempted to leverage translational discoveries to generate new and innovative immune therapies [15,16,17,18]. For instance, there is growing interest in targeting the unique immunosuppressive elements of the PDAC TME to enable immune recognition and antitumor effects [19]. Elements of the TME can allow PDAC to evade an effective antitumor immune response [19,20]. We herein review immunotherapy in the context of PDAC TME, as well as highlight data from several completed and ongoing clinical trials of immune therapies in the treatment of PDAC.

## 2. Tumor Microenvironment in Pancreatic Cancer

**Figure 1:**
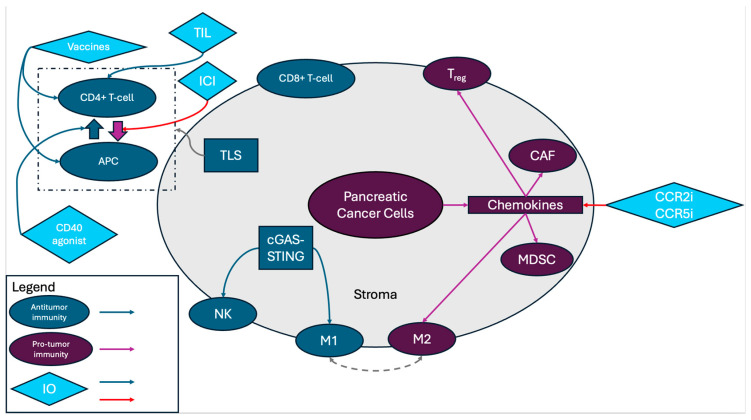
Schematic summarizing the key components of the tumor microenvironment and where immune therapies act within these pathways. Blue arrows from immune-oncology (IO) agents represent agonism whereas red arrows from IO agents represent antagonism. APC, antigen-presenting cell; TILs, tumor-infiltrating lymphocytes; ICIs, immune checkpoint inhibitors; TLSs, tertiary lymphoid structures; NK, natural killer cells; cGAS-STING, cyclic GMP-AMP synthase and Stimulator of Interferon Genes; M1, tumor-associated macrophage type 1; M2, tumor-associated macrophage type 2; Treg, regulatory T-cell; CAF, cancer-associated fibroblast; MDSC, myeloid-derived suppressor cells; CCR2i, CCR2 inhibitor; and CCR5i, CCR5 inhibitor.

A variety of factors contribute to the “cold” TME in PDAC [10,19]. These include proteins expressed or secreted by pancreatic cancer cells, the desmoplastic stroma characterized by cancer-associated fibroblasts and pancreatic stellate cells, the low TMB typical of PDAC, as well as the role of mutant KRAS (Figure 1).

### 2.1. Cancer Cell-Intrinsic Immune Evasion

The cellular component of the TME includes tumor cells, pancreatic stellate cells (PSC), and several types of immune cells, including T and B lymphocytes, natural killer (NK) cells, macrophages, neutrophils, fibroblasts, and myeloid-derived suppressor cells (MDSC) [20]. TILs are the ultimate target of immune checkpoint inhibitor (ICI) therapy. The programmed death (PD) receptor pathway and other immune checkpoints, such as cytotoxic T lymphocyte antigen 4 (CTLA4) and lymphocyte activation gene 3 (LAG3), normally lead to tolerance of self-antigens by limiting the proliferation of certain CD4+ T-cells [21]. Cancers can directly or indirectly induce this pathway through expression of programmed death ligand 1 (PD-L1) [21]. This interaction occurs at the level of the TME; the result of the immune checkpoint pathway is a predominantly exhausted TIL phenotype. Immune checkpoint inhibitors (ICIs) interfere with PD-1/PD-L1 signaling, re-invigorating exhausted T-cells. ICIs are the most widely studied immune agent in PDAC as their safety and efficacy have been well established in other cancers.

KRAS is the most common driver mutation in PDAC. Mutations in KRAS codon 12 or 13 lead to cancer cell-intrinsic production of CXCL1, a chemokine which recruits MDSC and stimulates MDSC to produce tumor necrosis factor (TNF) [22]. Co-mutation of TP53 with KRAS generally portends a very poor prognosis and is associated with a distinct mechanism of immune tolerance; the downstream interaction of TNF with the TNF receptor 2 leads to further immune tolerance by recruiting counter-regulatory immune cells [23,24,25]. PDAC also generates extracellular vesicles that may have several unique functions including the delivery of molecular signals to develop distant premetastatic niches and exerting influence on the TME [26]. Another important cancer cell-intrinsic mechanism is mitogen-activated protein kinase (MAPK) signaling via upregulation of p38, which leads to IL-1α production and recruitment of a dysregulated inflammatory response [27]. Immunotherapeutic agents targeting these pathways are in preclinical and early clinical trials.

### 2.2. Dysregulated Host Immune Response

Tumor-associated macrophages generally express two distinct phenotypes: an M1 phenotype, which has antitumor effects; and an M2 phenotype that downregulates cytotoxic activity and stimulates regulatory T-cells (T_reg_) [20,28]. Other counterregulatory T-cells, such as T_h_17 helper T-cells, work with T_reg_ to limit cytotoxic activity from the adaptive immune system [29]. Pancreatic stellate cells (PSCs) interact with pancreatic cancer cells to recruit the development of the dense desmoplastic stroma through a variety of pathways, including sonic hedgehog (SHH), CXCL1/2, and NF-kB [30,31]. PSCs also act to facilitate the epithelial–mesenchymal transition in pancreatic cancer cells, in which the loss of cell adhesion molecules and other characteristics of normal ductal epithelium facilitate invasion and metastasis [32]. The key downstream effector of the fibrotic extracellular matrix are cancer-associated fibroblasts (CAFs) [33]. Early-phase clinical trials have been conducted to examine ways to manipulate these pathways and are discussed below.

While the fibrotic TME of PDAC limits immune infiltration, the development of tertiary lymphoid structures (TLS) represents an intrinsic mechanism to overcome cancer-driven immune tolerance [34]. TLS are analogous to lymph nodes, whereby T and B lymphocytes interact with dendritic cells and other antigen-presenting cells to generate adaptive immunity [35]. The quantity and maturity of TLS have been reported to correlate with prognosis, as well as responses to immunotherapy [34,35,36].

Manipulation of the innate immune system is an increasing focus of investigation for multiple cancer types including PDAC [37]. The cyclic GMP-AMP synthase (cGAS) and Stimulator of Interferon Genes (STING) pathway have emerged as targets for modulation of innate immunity. The primary function of STING is as a cytosolic DNA sensor, leading to the activation of phagocytes such as macrophages and dendritic cells [38]. STING agonism has been demonstrated to overcome immune resistance in PDAC in animal models [39,40,41]. As the understanding of the innate immune system expands, this therapeutic approach may become an increasingly important modality for immunotherapy in PDAC.

## 3. Immune Checkpoint Blockade

Immune checkpoint inhibitors (ICIs) were the first immune therapies that were widely adopted to treat multiple cancer types. In light of the promising success for classically immunogenic tumors, several trials have examined ICIs for PDAC [42]. Results of one Phase II trial of single-agent ipilimumab, a CTLA-4 inhibitor, demonstrated no benefit in the treatment of metastatic PDAC [43]. In a separate Phase Ib study, ipilimumab in combination with gemcitabine was evaluated, and it demonstrated an objective response rate (ORR) of 16% [44]. Both trials included patients with treatment-refractory metastatic disease and did not select for susceptibility to ICIs. Subsequent trials have sought to define a role for ICIs in treatment sequencing for PDAC (Table 1).

### 3.1. ICIs in Microsatellite Unstable/Mismatch Repair-Deficient PDAC

Mechanistically, microsatellite instability (MSI) and DNA mismatch repair deficiency (dMMR) lead to a higher number of somatic mutations; this higher TMB leads to an immunogenic TME. The large number of tumor-associated antigens (TAAs) leads to increased CD4+ T-cell infiltration and activation, which contributes to abundant TILs with an exhausted phenotype that can be overcome by ICIs [56]. The KEYNOTE-158 trial noted a benefit of pembrolizumab (anti-PD-1) for a variety of non-colorectal microsatellite-unstable (MSI-H) or mismatch repair-deficient (dMMR) cancers, including PDAC [45]. Unfortunately, MSI-H/dMMR tumors comprise a small minority of patients with PDAC; in turn, the KEYNOTE-158 trial included only a small number of pancreatic cancers. A more recent multi-institutional retrospective cohort study of ICIs in 31 patients with MSI-H/dMMR PDAC demonstrated an ORR of 48.4% and median progression-free survival (PFS) of 26.7 months [57].

Lynch syndrome, which is characterized by germline mutation in one of five MMR proteins including MLH1, MSH2, MSH6, PMS2, and EPCAM predisposes patients to colorectal, endometrial, gastric, and pancreatic cancers, among others; most often, these are MSI-H tumors [58]. Interestingly, patients with Lynch syndrome and PDAC may be another subset of patients who benefit from ICIs. One retrospective cohort study demonstrated the efficacy of ICIs in patients with germline and somatic MMR mutation [59]. The authors noted that additional MMR/MSI testing in the setting of PDAC may help identify individuals who may be most likely to benefit from ICIs [59]. Since this subtype remains a small proportion of PDAC, however, most clinical trials have examined ICIs in patients with advanced PDAC regardless of MMR/MSI status.

### 3.2. ICIs in Metastatic PDAC

ICIs as a standalone therapy has largely been ineffective in advanced PDAC [60]. Therefore, most studies have combined ICIs with standard chemotherapy, radiation, or other experimental immune therapies. The rationale for adding ICIs to chemotherapy is the potentiation of ICI efficacy by chemotherapy’s effects on the TME, which include increased effector T-cell infiltration and macrophage class switching [11]. In a Phase Ib study, nivolumab (anti-PD-1) was added to gemcitabine and nab-paclitaxel (Gem-Nab) among patients with previously untreated locally advanced (LA) or metastatic PDAC [61]. Among 50 evaluable patients, the OS was 9.9 months and ORR was 18% [61]. In a separate Phase II trial (CCTG PA.7), the combination of durvalumab (anti-PD-1) and tremelimumab (anti-CTLA-4) in addition to Gem-Nab was evaluated in 119 patients with previously untreated LA or metastatic PDAC [62]. The primary endpoint of median OS was not met (9.8 vs. 8.8 months); the PFS was also similar between the two cohorts [62]. Other similar trials are ongoing, such as a Phase Ib/II study of Gem-Nab with or without toripalimab (anti-PD-1) [63]. In addition, novel delivery methods are being investigated to overcome the fibrotic tumor stroma [64,65]. KN046, a bispecific antibody targeting both PD-1 and CTLA-4, has demonstrated promising results in combination with Gem-Nab, with an ORR pf 56% in patients with LA or metastatic PDAC; a larger trial is also ongoing (NCT05149326) [66].

The CheckPAC trial evaluated nivolumab with or without ipilimumab in addition to stereotactic body radiotherapy (SBRT) among patients with metastatic PDAC who progressed on first-line standard therapies [67]. In this Phase II trial including 84 patients, the ORR was 14% for the dual ICI group [67]. Despite the presence of metastatic disease, SBRT was employed to elicit the expression of the damage-associated molecular patterns, which may lead to a more robust immune response [14]. However, in the CheckPAC trial, there was no significant change in the TME with SBRT [67]. Despite preclinical evidence, other clinical trials have similarly failed to demonstrate an immunologic or clinical benefit of using radiation to potentiate the efficacy of ICIs [48,68].

### 3.3. Combination Immunotherapy in Metastatic PDAC

Other trials have combined ICIs with other targeted therapies, including poly-ADP ribose polymerase (PARP) inhibitors such as olaparib for platinum-sensitive PDAC, vascular endothelial growth factor (VEGF) inhibitors such as cediranib, the MEK inhibitor trametinib for KRAS mutant PDAC, and the IL-6 inhibitor tocilizumab—all with very poor response rates (Table 2) [48,52,69,70,71,72]. One study that examined maintenance ICIs and niraparib among patients who had stable disease on platinum chemotherapy demonstrated a 6-month PFS of 59% for individuals receiving ipilimumab with niraparib [52]. Given the aggressive nature of PDAC, these findings may be attributed to a select cohort who had already demonstrated disease stability over time. Ultimately, no study of ICIs as a standalone immune therapy, even when combined with chemotherapy, radiation, or targeted agents, has demonstrated clinically significant responses or survival for metastatic PDAC. Marked intratumoral heterogeneity, as well as the unique immune profiles of metastatic niches, make it unlikely that the single mechanism of checkpoint inhibition will be sufficient to overcome immune evasion and resistance in metastatic PDAC [73,74].

### 3.4. Neoadjuvant ICIs

Many patients with localized PDAC, especially in the setting of borderline resectable (BR) or LA, receive neoadjuvant therapy [75]. The most common neoadjuvant regimens are 5-fluorouracil, leucovorin, irinotecan, and oxaliplatin (FOLFIRINOX); gemcitabine and nab-paclitaxel (Gem-Nab); and gemcitabine-based chemoradiation (Gem-RT) [5,6,7,8]. Total neoadjuvant therapy (TNT), consisting of chemotherapy and chemoradiation prior to surgery, is another evolving strategy. In theory, ICIs may be more useful in the neoadjuvant setting compared with the metastatic setting because of the opportunity to entrain an adaptive immune response to tumor-associated antigens [16,76]. In highly immunogenic tumors, neoadjuvant ICIs often achieve a major pathologic response, typically defined as >70% or >90% tumor necrosis, or even a pathologic complete response [77,78]. While such dramatic responses to ICIs are not anticipated in PDAC, investigators have assessed whether adding ICIs to neoadjuvant chemotherapy can improve local downstaging.

The CISPD-4 study evaluated neoadjuvant FOLFIRINOX with or without a PD-1 inhibitor for BR/LA PDAC [46]. A partial response, assessed by preoperative imaging, occurred in 36% in the PD-1 group versus 13% for chemotherapy only. Approximately one half of patients in each group underwent surgical resection; of note, among patients with LA, the PD-1 group achieved a conversion to resection more often (48% vs. 37%). In addition, the PD-1 group was more likely to have a margin-negative resection (87% vs. 70%) [46]. Long-term survival data were not available but are anticipated. Two other ongoing trials are evaluating the addition of commonly used ICIs to modified FOLFIRINOX as a neoadjuvant therapy, with one study using pembrolizumab (NCT05132504) and the other, the NEO-IMPACT trial, using durvalumab (NCT06094140).

#### 3.4.1. Neoadjuvant ICIs with Radiation

Radiation modulates the TME and improves the efficacy of ICIs in preclinical models, but this strategy has yielded mixed results in clinical trials. In a multicenter randomized trial, the addition of pembrolizumab to neoadjuvant capecitabine-based chemoradiation demonstrated no improvement in the OS nor in immune infiltration [79]. One Phase II study of sintilimab (anti-PD-1) with Gem-RT in LA PDAC suggested a benefit in adding ICIs to chemoradiation. The ORR was 32% versus 20% for Gem-RT alone; in addition, the median OS (22 months vs. 16 months) and PFS (12 vs. 10 months) were improved [49]. Conversion to surgical resection was not reported [49].

Notably, the Alliance A021501 trial demonstrated no OS benefit of neoadjuvant chemoradiation when added to FOLFIRINOX [80]. Similarly, results from the PRODIGE-44 trial of FOLFIRINOX with or without chemoradiation demonstrated no improvement in the survival or margin-negative resection with TNT (NCT02676349). These results may influence a shift away from TNT except in the setting of LA/unresectable PDAC.

#### 3.4.2. ICIs with Total Neoadjuvant Therapy

A prospective cohort study evaluated Gem-Nab with or without camrelizumab (anti-PD-1) and radiation. The median OS was 22 months vs. 19 months for chemotherapy alone; however, this study was only able to evaluate the combination of ICIs and radiation compared with chemotherapy, rather than the effect of ICIs alone [50]. A Phase II trial of tislelizumab (anti-PD-1) combined with Gem-Nab and SBRT demonstrated an ORR of 60% and a 12-month OS of 72% [51]. In a different trial, nivolumab and losartan (an angiotensin converting enzyme inhibitor that may potentiate chemotherapy efficacy), were combined with FOLFIRINOX and SBRT [81]. The primary endpoint of improved OS was not met, but the full results are not yet available [81].

#### 3.4.3. Perioperative Considerations

Pancreatic surgery can be associated with a high incidence of complications, making neoadjuvant chemotherapy an appealing strategy to ensure delivery of systemic therapy in the preoperative setting. Cytotoxic chemotherapy is rarely associated with toxicity that delays or prohibits subsequential pancreatic resection. ICIs can, however, have substantial adverse events that require special attention in the perioperative period, including endocrinopathies such as adrenal insufficiency, or in other autoimmune conditions such as pneumonitis, hepatitis, or colitis requiring corticosteroid therapy. These immune-related adverse events (irAE) can delay or complicate an already complicated procedure such as pancreatoduodenectomy [82]. Combination immune therapy also increases the risk of irAE, although the development of irAE correlates with the response to therapy [83]. Proceeding trials of neoadjuvant ICIs will need to examine the toxicity associated with this therapy relative to surgical considerations.

#### 3.4.4. Personalized Neoadjuvant Therapy

The marginal increase in response rates, conversion to resection, and margin-negative resection seen in neoadjuvant ICIs and chemotherapy trials suggest that there is a subset of patients who would benefit the most from ICIs. Testing on biopsy samples, such as PD-L1 staining, MMR and MSI status, and TMB, could be used to guide targeted neoadjuvant therapy, but this would still represent a small proportion of PDAC patients. Precision-guided neoadjuvant treatment paradigms are under investigation [84]. Newer molecular techniques may provide more integrative and holistic insights into molecular drivers and immunophenotypes in PDAC [85,86]. Four distinct transcriptomic profiles—antitumor immune, protumor immune, stromal and angiogenesis processes, and epithelial–mesenchymal transition—were integrated to describe four distinct immunophenotypes: immune-enriched, immune-enriched and fibrotic, immune-depleted, and fibrotic [87]. Future trials should evaluate precision neoadjuvant therapy, which may include ICIs for susceptible tumors, based on these distinct TME phenotypes.

## 4. Emerging Immune Targets

### 4.1. T-Cell Interactions Beyond PD-1 and CTLA-4

**Figure 2:**
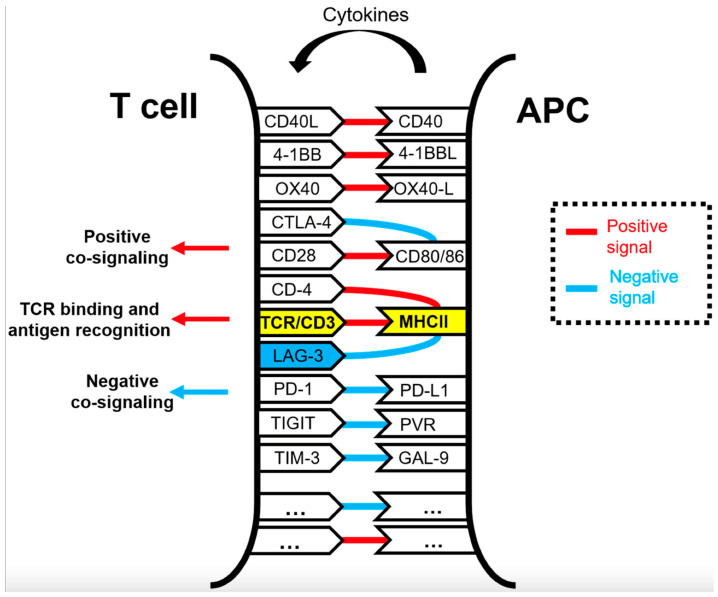
Immune checkpoints (red) and costimulatory (blue) interactions between antigen presenting cells and T-cells. Adopted from Chocarro et al., 2021 (open access) [88].

In addition to the interaction between PD-1/PD-L1 and CTLA-4 with CD80/86, additional immune checkpoint interactions have been described and are the focus of novel immune therapies (Figure 2). The lymphocyte activation gene 3 (LAG3) receptor located on CD4+ T-cells is involved in the major histocompatibility complex (MHC) class II interaction with the T-cell receptor (TCR) [88]. The combination of PD-1 and LAG-3 inhibition is already used in melanoma and has efficacy in MSI-H/dMMR colorectal cancer [89,90]. In PDAC, LAG3 and other checkpoints such as TIGIT are being investigated [91].

The CD40 and CD40 ligand (CD40L) interaction between antigen-presenting cells (APCs) and T-cells functions as a costimulatory signal rather than an immune checkpoint. In preclinical studies, CD40 agonism activates cross-presenting dendritic cells, induces macrophage class switching toward an M1 phenotype, decreases fibrosis in the tumor stroma, and triggers antitumor T-cell responses [92,93]. Selicrelumab, a CD40 agonist, demonstrated changes in the PDAC TME when used in the neoadjuvant setting [94]. The Phase II PRINCE trial evaluated Gem-Nab with or without nivolumab and the CD40 agonist sotigalimab [47]. In previously untreated patients with metastatic PDAC, the nivolumab and chemotherapy group had a one-year OS of 58%, which was better than the OS of 41% noted in the group of patients treated with combination sotigalimab/nivolumab/chemotherapy [47]. Different combinations of immune checkpoint blockade with costimulatory agonism are under preclinical investigation.

### 4.2. Targeting Chemokines

A rapidly expanding body of preclinical evidence is actively being translated into novel immune therapies in PDAC, with efforts focused on modulating non-lymphocyte components of the TME such as cancer cell-intrinsic chemokine signaling, CAFs, MDSCs, and innate immunity. Thus far, the earliest completed trials have evaluated agents that interfere with chemokine signaling, thereby limiting the recruitment of T_reg_ and M2 macrophages. The chemokines CCR2 and CCR5 bind to various CXCL molecules to form heterodimers that promote lymphocyte and macrophage chemotaxis [95]. A Phase I study combining a CCR2/CCR5 inhibitor with FOLFIRINOX in BR/LA PDAC reported an ORR of 49% [96]. Preclinical studies have demonstrated that CCR2/CCR5 are upregulated with anti-PD-1 administration, which may represent an escape mechanism of ICI resistance [97]. Therefore, clinical trials combining CCR2/CCR5 inhibition with ICIs are ongoing [98]. The COMBAT trial evaluated nanoliposomal irinotecan and 5-fluorouracil (the NAPOLI-1 regimen) with pembrolizumab and motixafortide, a CXCR4 inhibitor [53]. Among patients with metastatic PDAC who progressed on gemcitabine, this combination yielded an ORR of 21% [99]. Additional trials targeting CXCL2, semaphorin 4D, and MDSC function are ongoing [22,100].

### 4.3. Innate Immunity

The contribution of the innate immune system to the PDAC TME has recently been characterized. As the innate immune system is crucial for host defenses against infection, agonism of the cGAS-STING pathway has been studied using alternate drug delivery methods such as injectable nanoparticles, whereas systemic delivery could carry a high risk of off-target toxicity [39]. Clinical data for cGAS-STING agonism in PDAC are not yet mature. The effect of STING agonism on the PDAC TME is context-dependent, in part due to tumor heterogeneity, so combination with other therapies is likely required [101]. Combination with ICIs could also obviate the need for nanoparticle delivery, as STING agonism increases the expression of immune checkpoint ligands in preclinical models, suggesting an opportunity for synergistic antitumor immunity with STING agonism and the immune checkpoint blockade [102].

## 5. Cancer Vaccines

Most cancer vaccines have had poor efficacy in most solid tumors, especially in the advanced setting [103,104]. Few trials have examined neoadjuvant vaccine strategies in pancreatic or hepatobiliary cancers, with a few notable exceptions [36,105]. Vaccines are generally well tolerated but have limited efficacy unless combined with ICIs or other therapies [106]. This therapy can involve either a personalized vaccine against unique TAAs or a more generic vaccine against prespecified TAAs that is commonly expressed in each cancer type. Vaccines can be delivered as peptide neoantigens, autologous cells, allogeneic cells, or messenger RNA (mRNA) [107,108,109]. In contrast to immunogenic cancers, PDAC has a lower repertoire of targetable TAAs, but there are up to ten consistent neoantigenic targets including the transcription products of mutant KRAS and TP53 [110]. Peptide vaccines have demonstrated only modest clinical benefit, even for cancers with characteristic and prolific neoantigens such as melanoma and HER2-overexpressing breast cancer [111,112].

The earliest vaccine in PDAC was GVAX, an allogeneic genetically engineered tumor cell product designed to secrete granulocyte-macrophage colony stimulating factor (GM-CSF) [113]. GVAX has limited efficacy as a standalone agent or when combined with ICIs [113,114]. Several trials have evaluated combinations of other immune therapies with GVAX, including a Listeria species as a modulator of the gut and tumor microbiome, CD137, and CCR2/CCR5 inhibitors [98,105,115]. The immunologic correlative data for GVAX are strong; GVAX is able to increase tertiary lymphoid structures that have been associated with improved survival among patients with PDAC [34,35,116]. Unfortunately, data from clinical trials have failed to demonstrate a meaningful survival advantage with GVAX. When combined with nivolumab and SBRT for a metastatic PDAC refractory to first-line therapy, GVAX has been reported to have a major pathologic response of 35% and one pathologic complete response [117]. Hyper-Acute-Pancreas, or algenpantucel-L, is also an allogeneic engineered pancreatic cancer cell product, in which cells are engineered to express murine alpha-(1,3)-galactosyltransferase. A Phase III trial of chemotherapy and chemoradiation with or without algenpantucel-L for BR/LA PDAC demonstrated no significant improvement in OS, PFS, or conversion to resection [118].

### 5.1. Cellular Vaccines

Dendritic cell vaccines streamline the delivery of an antigenic payload to the adaptive immune system. An off-the-shelf or personalized (e.g., tumor lysate) neoantigen array is loaded into autologous dendritic cells grown from peripheral blood mononuclear cells. The DC are then delivered as a vaccine to present the neoantigens in vivo to CD4+ T-cells to elicit an antitumor immune response [119]. DC vaccines have been studied in melanoma and glioblastoma [120,121]. A Phase I study of adjuvant autologous DC pulsed with allogeneic tumor lysate demonstrated the feasibility of this strategy for PDAC, although outcome data were limited [122]. Preclinical studies have examined the addition of irreversible electroporation (IRE) to DC vaccine therapy, with the rationale that IRE disrupts the desmoplastic stroma and causes the release of neoantigens that DC can present to T-cells, but clinical results have been disappointing [123].

### 5.2. mRNA Vaccines

A quantum leap in the development of mRNA vaccines occurred during the COVID-19 pandemic, coinciding with several trials of mRNA vaccines in various cancers. The personalized mRNA vaccine autogene cevumeran was evaluated in a Phase I trial with atezolizumab (anti-PD-L1) and chemotherapy among patients with resected PDAC. The vaccine is immediately manufactured upon surgical resection and personalized to the individual’s TAAs recovered from the pathologic specimen. Among the 16 patients who received upfront surgery followed by adjuvant autogene cevumeran, atezolizumab, and FOLFIRINOX, 8 had neoantigen-specific T-cell responses. At 18 months median follow-up, these immune responders had a significantly improved median recurrence-free survival compared with the non-responders (not reached vs. 13.4 months) [54]. This regimen is under investigation in the Phase II IMCODE003 trial (NCT05968326).

## 6. Adoptive Cell Therapies

Cellular therapy includes T-cell receptor (TCR) engineering, chimeric antigen receptor T-cells (CAR-T), and TIL therapy. These therapies target specific antigens, theoretically minimizing off-target toxicity, yet the necessity for antigen specificity may limit widespread use given the antigenic diversity inherent to solid tumors including PDAC. Although the number of neoantigens expressed by PDAC is lower than in other solid tumors, there is still considerable antigenic diversity between patients, exemplified by the various types of KRAS mutations commonly seen in PDAC [124].

### 6.1. T-Cell Receptor Engineering

TCR-engineered T-cell therapy involves conjugating a tumor-specific TCR to CD3, which activates cytotoxic and helper T-cells [125]. This process requires human leukocyte antigen (HLA)-A restriction because of the specificity of TCR-MHC binding. One case report has demonstrated the feasibility of TCR therapy targeting a KRAS G12D mutation in PDAC; the patient had a partial response at sites of visceral metastasis, and the response was ongoing at 6 months [124]. An HLA-C-restricted, TCR-engineered autologous T-cell product targeting KRAS G12D is under investigation in a clinical trial (NCT06218914). An intriguing hybrid strategy, using autologous, non-engineered T-cells targeting prespecified TAAs (PRAME, SSX2, MAGEA4, NY-ESO-1, and Survivin) given with autologous DC, was evaluated in the TACTOPS trial [55]. Among 13 selected patients with LA or metastatic PDAC with stable disease on first-line therapy, one had a complete clinical response and 8 had partial responses [55].

### 6.2. Chimeric Antigen Receptor T-Cell Therapy

CAR-T is an antigen-specific cellular therapy that requires nonmyeloablative lymphodepletion followed by the delivery of genetically modified T-cells expressing a chimeric TCR that recognizes a TAA and costimulates T-cell activation [126]. The first successful use of CAR-T was in B-cell malignancies by targeting CD19 [127]. Using CAR-T to “make solid tumors liquid” has not yet been fully established as it requires targeting a specific TAA. CAR-T currently requires a viral delivery system to insert the gene for the desired TCR into the host T-cell genome, which leads to high cost and the potential for mutagenicity, although newer CAR-T products may be created using non-viral gene editing methods [128]. Preclinical feasibility studies have been published for CAR-T products with various mechanisms, including CAF inhibition and tumor-associated macrophage depletion, among others [129,130,131]. Clinical data are limited to case reports [132].

### 6.3. Tumor-Infiltrating Lymphocytes

TIL therapy is distinct from TCR engineering and CAR-T in that it delivers pluripotent TAA-specific T-cells. Tumor-reactive TILs are TAA-specific but not restricted to a limited number of TAAs. TIL therapy requires a labor-intensive process of nonmyeloablative lymphodepletion, operative metastasectomy to obtain tumor tissue, culturing and activating the TILs, as well as infusing the TIL product along with IL-2 as an immune adjuvant. The delivery method carries significant toxicity, as would be expected with therapeutic doses of IL-2; in fact, there was an associated 7.5% mortality rate in melanoma trials [133]. TIL therapy for PDAC is being studied in two Phase II basket trials (NCT01174121, NCT03610490) [134]. Patients who respond to TIL therapy typically have durable responses, but the short-term toxicities can be substantial and response rates would likely be less favorable in PDAC compared with melanoma.

## 7. Discussion and Future Directions

Early trials of immunotherapy in PDAC have been conducted in patients with metastatic disease refractory to prior lines of therapy and, unsurprisingly, yielded discouraging results. At advanced stages of disease, intra-tumor and inter-tumor heterogeneity in somatic molecular profiles, as well as the immune TME, will likely limit any meaningful effect of immune-based therapies [135]. Except in dMMR/MSI-H or PD-L1-positive PDAC, ICIs should not be used as a “last resort” option as ICIs have been associated with hyperprogressive disease in heavily pretreated metastatic PDAC [136]. ICIs and vaccines, alone or in combination, may have a more beneficial role in the neoadjuvant or adjuvant setting with a shift in treatment sequencing toward a neoadjuvant approach as it may be more practical to evaluate immunotherapy in the preoperative setting. Furthermore, there are rationale and clinical data to support neoadjuvant rather than adjuvant immunotherapy, as sustained adaptive immune responses may be superior when ICIs are delivered with the tumor in situ. Thus, the niche for ICIs and vaccines in the PDAC treatment armamentarium may be in preventing recurrence rather than in treating locally or expecting a measurable response to measurable disease [137,138]. The optimal combination of treatment modality and sequence has not yet been elucidated in a Phase III trial (Table 2).

Local response to neoadjuvant therapy can improve the likelihood of a margin-negative resection. In some rare cases, neoadjuvant therapy reduces the tumor volume to make an anatomically unresectable tumor resectable; more commonly, the radiographic response is minimal but, at final pathology, the margin is fibrotic and lacking any viable tumor cells. Based on current clinical trial data, neoadjuvant immunotherapy is unlikely to achieve clinical responses by RECIST criteria because it induces tumor necrosis but not a significant reduction in tumor volume. Immunotherapy can increase the degree of pathologic response, which, in PDAC, may translate to a survival benefit [139]. Therefore, low response rates by RECIST criteria in the advanced setting do not necessarily convey a lack of efficacy relative to survival, particularly in view of the potential for entraining cellular antitumor immunity.

While PDAC has a characteristically immunosuppressive TME, recent advances in understanding the mechanisms underlying this immunophenotype are paving the way for novel immune targets. Therapies targeting chemokines, CAFs, MDSCs, and other TME-resident immune cells are on the horizon. A myriad of additional targetable mechanisms are being discovered, although a combination approach is likely needed to achieve a clinical benefit from TME-modulating therapies.

Advances in molecular techniques enable more accurate characterization of immune and molecular phenotyping of PDAC that can translate to personalized therapy decisions. Perhaps the first step will be implementation of molecular and immune phenotyping from biopsies, which could be used to select neoadjuvant therapy for localized disease or first-line therapy for metastatic disease [87]. Among the subset of patients with PDAC who have an immune-enriched phenotype, adding ICIs to chemotherapy may improve prognosis [140]. Personalized therapy will also allow patients with immune-privileged and fibrotic PDAC to forego the cost and irAE associated with ICIs or other immune therapies. The initial success of adjuvant personalized mRNA vaccines to prevent recurrence represents a glimmer of hope for immunotherapy in PDAC. Disease recurrence is an ominous event and often heralds progressive metastatic disease, and treatment options are typically limited. The ability to prevent recurrence by harnessing the effectiveness and durability of adaptive immunity could represent a paradigm shift in the treatment of localized PDAC.

Until larger trials are completed, the current role of immunotherapy in localized PDAC is limited to the small number of patients with dMMR/MSI-H tumors. In metastatic PDAC, ICIs can be considered as a component of maintenance therapy among patients with stable disease. Future clinical and translational trials, particularly in the neoadjuvant setting, will hopefully lead to improved understanding of the role of immunotherapy in the treatment of patients with PDAC.

## Figures and Tables

**Table 1:** Summary of the clinical trials cited herein with their reported results.

	Adjuvant	Neoadjuvant	Metastatic	Positive Trials
ICI monotherapy	*	*	*	KEYNOTE-158 [45]
ICIs + chemotherapy		+	+ − −	CISPD-4 [46], PRINCE [47]
ICIs + radiation		+ + −	+ − −	Pembro + SBRT + Trametinib [48]; Sintilimab + Gem-RT [49]
ICIs + TNT		+ + −		Camrelizumab + TNT [50]; Tislelizumab + TNT [51]
ICIs + targeted therapy			+ − −	Ipi + PARPi [52]
ICIs + other IO			+ − − − − − − − − −	COMBAT [53]
Vaccine	+	−	+ −	Autogene cevumeran [54], TACTOPS [55]

+ Trials where the experimental arm met the primary endpoint or were considered favorable. − Trials where the primary endpoint was not met, or response rates were <20%. * Mismatch repair-deficient or microsatellite unstable tumors.

**Table 2:** Completed clinical trials of immune checkpoint inhibitors in metastatic PDAC.

First Author	PMID	Trial Name	ICI	Other Immune Agent	Other IO Category	Chemo/RT	Population
Chen	36592507	TRIPLE-R	Ipi + Nivo	Tocilizumab	Anticytokine	SBRT	PD on 1st line
Enzler	38422585		Nivolumab	CBP501	Calmodulin binding	Cisplatin	PD on 2 prior lines
Padron	35662283	PRINCE	Nivolumab	Sotigalimab	CD40 agonist	Gem-Nab	Previously untreated
Bockorny	34253578	COMBAT/KEYNOTE-202	Pembro	Motixafortide	CXCR4 blockade	Nal-Iri + 5FU (NAPOLI-1)	Progressed on gem
Wang-Gillam	36228156		Pembro	Defactinib	FAK inhibitor	Gemcitabine	PD on 1st line, or SD after Gem-Nab
Voisin	38502104	CATRIPCA	Pembro	Xevinapant	Inhibitor of Apoptosis Protein (IAP)		
Melisi	33688022		Durvalumab	Galunisertib	TGFB-R1		
Lemech	36634920		Nivolumab	Pixatimod	TLR9 agonist		
Ko	36940261	MORPHEUS	Atezolizumab	PEGPH20	TME other	Compared	
Tsujikawa	32273276		Nivolumab	GVAX	Vaccine	No; Listeria	PD on 1st line
Hernando-Calvo	38960798	DAPPER	Nivolumab			Cediranib or olaparib	
Renouf	36028483	CCTG PA.7	Durvalumab + Tremelimumab			Gem-Nab	Previously untreated
Wainberg	32554514		Nivolumab			Gem-Nab	Previously untreated
Reiss	35810751		Nivo or Ipi			PARPi	SD on platinum chemo
Chen	35476508	CheckPAC	Nivo +/− Ipi			SBRT	PD on 1st line
Xie	31996388		Durvalumab + Tremelimumab			SBRT	PD on 1st line
Zhu	35240087		Pembro			SBRT + Trametinib or gem	Locally recurrent and KRASmut and PD-L1+

ICI, immune checkpoint inhibitor; Ipi, ipilimumab; Nivo, nivolumab; Pembro, pembrolizumab; IO, immuno-oncology; Gem-Nab, gemcitabine and nab-paclitaxel; and SBRT, stereotactic body radiotherapy.
